# Selective serotonin reuptake inhibitors and suicidality in children and young adults: analyses of pharmacovigilance databases

**DOI:** 10.1186/s40360-023-00664-z

**Published:** 2023-03-31

**Authors:** Diana Dubrall, Stefanie Fekete, Sarah Leitzen, Lena Marie Paschke, Marcel Romanos, Matthias Schmid, Manfred Gerlach, Bernhardt Sachs

**Affiliations:** 1 Institute for Medical Biometry, Informatics and Epidemiology, University Hospital of Bonn, Bonn, Germany; 2 Federal Institute for Drugs and Medical Devices (BfArM), Research Division, North Rhine-Westphalia, Bonn, Germany; 3 Department of Child and Adolescent Psychiatry, Psychosomatics and Psychotherapy, University Hospital of Würzburg, Centre for Mental Health, Margarete-Höppel-Platz 1, 97080 Würzburg, Germany; 4 Department of Physics, Chemistry and Pharmacy, University of Southern Denmark, Odense, Denmark; 5 Central Research Institute of Ambulatory Health Care in Germany, Berlin, Germany; 6 Department for Dermatology and Allergy, University Hospital Aachen, Aachen, Germany

**Keywords:** Antidepressants, Depression, Suicide, Child, Adverse drug reaction

## Abstract

**Background:**

Since the warnings by the United States (US) and European regulatory authorities in 2004 and 2005 it had been discussed whether there is some link between selective serotonin reuptake inhibitors (SSRIs) and suicidality in the pediatric population. The aim of our study was to describe trends and patterns in spontaneous reporting data referring to suicidality in children, adolescents and young adults treated with SSRI after the warnings.

**Methods:**

Descriptive analyses of reports for 0–24 year olds referring to suicide/suicidal ideations, self-harms and overdoses with SSRIs reported as suspected submitted to the US (FAERS) and the European (EudraVigilance) adverse drug reaction databases until 2019 were performed. The causal relationship was assessed in accordance with the WHO criteria for the European reports. For Germany, prescription data for SSRIs were provided and reporting rates (number of reports/number of prescriptions) were calculated for the reports with possible causal relationship (so called “confirmed reports”).

**Results:**

Since 2004, the number of reports referring to suicide/suicidal ideations, self-harm and overdoses increased steadily in the US and EU. However, only a slight increase was seen for the confirmed EU reports. After 2008, the proportion of reports informing about suicidal ideations increased, while the proportion of fatal suicide attempts decreased. Reporting rates were higher for females and adolescents (12-18 years).

**Conclusions:**

Our results demonstrate the importance of further monitoring suicidality in 0–24 year olds treated with SSRI in order to recognize suicidality early avoiding fatal suicide attempts. The higher reporting rates for females and adolescents should be further investigated.

**Supplementary Information:**

The online version contains supplementary material available at 10.1186/s40360-023-00664-z.

## Background

In 2004 [1] and 2005 [2], the United States of America and European regulatory authorities issued public health warnings based on data from randomized controlled trials (RCTs) suggesting that the risk of suicidal thinking and behavior (suicidal attempts, preparatory behavior and suicidal ideations) following treatment with antidepressants, including selective serotonin reuptake inhibitors (SSRIs), were two times higher compared to placebo in children and adolescents [3]. Similar results were reported by others [4]. However, no suicides were recorded in these clinical trials. Based on respective analyses [5, 6], the number of adverse event reports referring to SSRIs and suicidality increased in the United States (US) and United Kingdom (UK) before the warnings. To the best of our knowledge, there were no analyses of spontaneous adverse drug reaction (ADR) reports during the warning period or afterwards for the US, the European Union (EU) and for Germany.

The suicidality warnings were also followed by investigations of their impact on trends and patterns of antidepressant use and variation in suicide rates. For example, there was a decrease in the prescribing rates of antidepressants in the US [7, 8] and some European countries [9–12] within two years. As in the US and other European countries, there was a slight decrease in antidepressant prescriptions immediately after the warnings in Germany [11, 12]. In children, the trend continued with a further decrease in the post-warning period, however, in adolescents SSRI prescriptions increased between 2006 and 2012 [11].

Risk factors that are assumed to increase the occurence of suicidality are, among others, an age of 10–24 years, depression and previous self-harm or suicide attempts [13, 14].

The aim of this study was to analyze trends and patterns in spontaneous reports of ADR referring to SSRIs and describing suicide/suicidal ideations, self-harm or overdoses in children, adolescents and young adults. Therefore, we systematically analyzed the Food and Drug Administration (FDA) Adverse Event Reporting System (FAERS) for the US and the European ADR database EudraVigilance for the EU during the period from 1978 to 2019. First, we hypothesized that the regulatory warnings may also have impacted on the annual number of reported suicide/suicidal ideations, self-harm incidents or overdoses associated with SSRIs in the warning period. Secondly, we wanted to analyze if the number of ADR reports increased again several years after the warnings in the US and the EU. In FAERS, the data access is restricted and, among others, information about patient history or narratives are not available, thus, an individual case assessment could not be done. In order to strenghten the analyses, the causal relationship was assessed in the reports from the EU and were analysedin order to identify reported characteristics and associated factors. Finally, we considered the number of reports from Germany in relation to the number of SSRI prescriptions and the number of suicides in the German population because no exact SSRI prescription data for children, adolescents and young adults were available for the whole EU.

## Methods

### Data sources and identification of cases

**Fig. 1:**
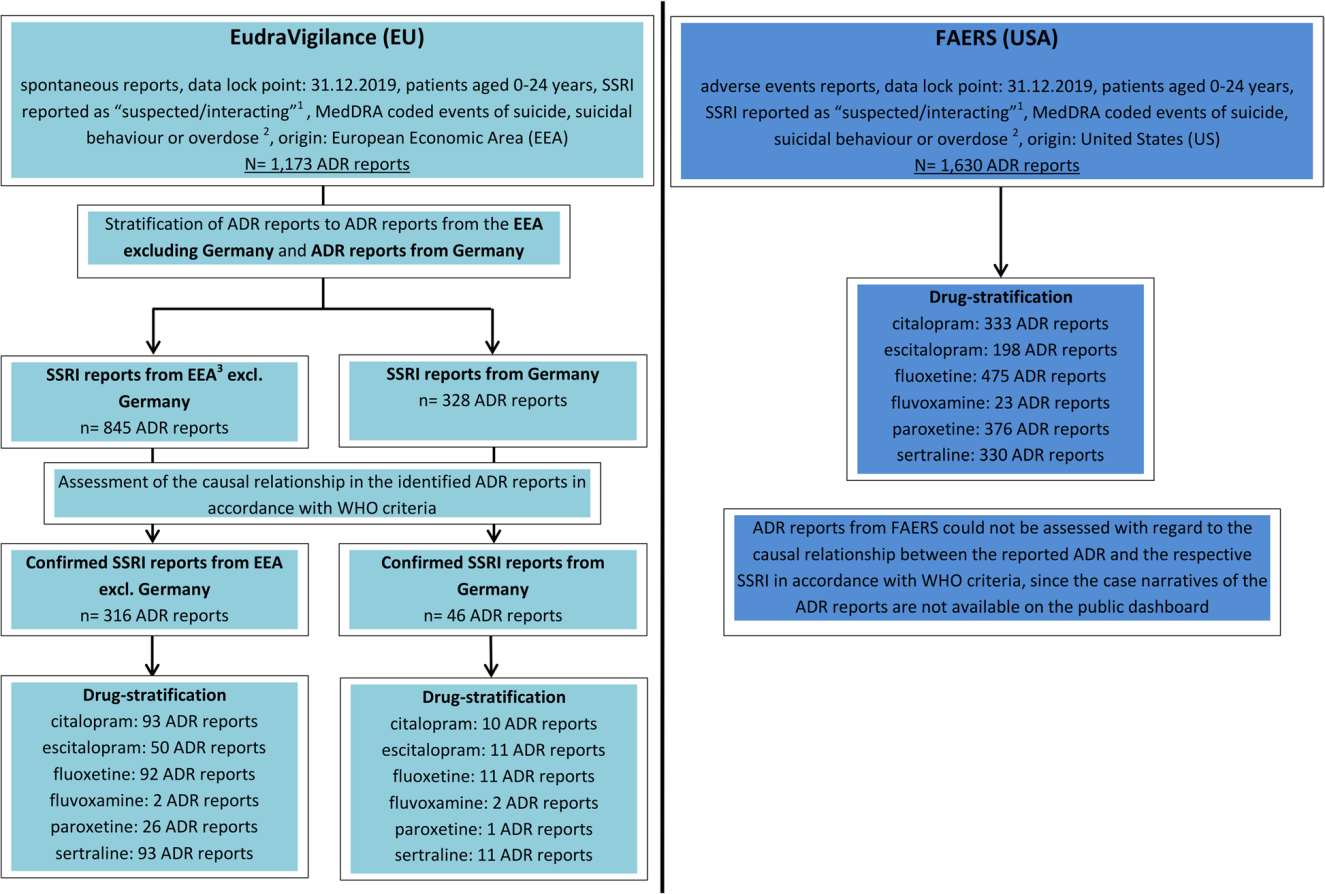
Flowchart. Research strategy for identification of intentional suicide/suicidal behaviour and overdose reports referring to selective serotonin reuptake inhibitors (SSRIs) in the European ADR database EudraVigilance and the Food and Drug Administration (FDA) Adverse Event Reporting System (FAERS). ADR: Adverse drug reaction; EEA: European Economic Area

In both databases, FAERS [15] and EudraVigilance [16], those ADR reports received up to 2019 for 0–24 year olds were extracted, in which a suicide/suicidal ideations, intentional self-harm or overdose in accordance with MedDRA terminology [17] and one of the SSRIs listed in ATC N06AB were coded (see Fig. 1).

The same research criteria were used to identify any other ADR reports to SSRI in EudraVigilance except for those identified referring to suicide/suicidal ideations, intentional self-harm or overdoses (see Data S1, available online).

In accordance with the legal definition, an ADR is a noxious, unintended response to a medicinal product within or outside its terms of marketing authorization. The latter has applied since 2012 and describes ADRs caused by medication errors, suicide/self-injury behaviours and intentional overdoses [18].

For our analyses, the following preferred terms of MedDRA terminology [17] were used for identification of ADR reports referring to suicide/suicidal ideations, intentional self-harm or overdoses: assisted suicide, completed suicide, intentional overdose, intentional self-injury, deliberate poisoning, suicide attempt, suspected suicide, suspected suicide attempt, columbia suicide severity rating scale abnormal, suicidal depression, self-injurious ideation, suicidal behavior, suicidal ideation and suicide threat were queried.

Note that the two databases did not overlap, as the analyses in FAERS and EudraVigilance were restricted to reports from the US and the EU, respectivly. Reporting obligations and data accessability of both databases can be found elsewhere [19].

To asses whether reported suicidality is associated with SSRI treatment, an individual case assessment with regard to the causal relationship between the reported reaction and the suspected SSRI was performed in accordance with the WHO criteria [20] for the EU reports. In addition, we examined if the respective MedDRA terms were coded.

- (i) as an ADR related to the respective SSRI therapy (in the following: ADR reports referring to suicidality) or.
- (ii) as a self-poisoning event in suicidal purposes without any evidence of regular SSRI therapy.

Finally, only reports with a certain, probable or possible causal relationship and reports referring to suicidality as an ADR were considered for further analysis (*n* = 362 “confirmed EU reports”). Furthermore, we identified all reports from Germany in order to set them in context with the number of drug prescriptions and the number of suicides in Germany (*n* = 46, 12.7%, “confirmed reports from Germany”).

The anonymized prescription data for the analyzed SSRIs for patients aged 12–24 years were provided by the Research Institute for Ambulatory Health Care in Germany for the period 2009–2019. The prescription data reflect the number of drug prescriptions for patients with statutory health insurance dispensed in a German pharmacy. It must be considered that the number of prescribed drugs may not represent the drugs that were actually taken (according to the physician's instructions).

The annual numbers of fatal suicide attempts for persons younger than 25 years in Germany were taken from the Federal Statistical Office in Germany [21]. The search was performed in December 2020.

### Data analyses

In both databases, we analyzed the number of ADR reports per year for all SSRIs and subdivided by drug substances descriptively. The period investigated was further divided into pre-warning (1978–2003), warning (2004–2008) and post-warning (2009–2019) periods. The time of the warning-period that was chosen includes the timing of the U.S. (2004) [1] and European (2005) [2] warnings and the extension of the warning to young adults (2007) [22].

During the individual case assessment, the ADR reports from EudraVigilance were assigned to one or more of the four categories: suicide, suicidal ideations, self-injury, and overdose (more than one assignment was possible). Whenever possible, data was retrieved with regard to the:

(i) Demographical parameters of the patients.
(ii) Respective SSRI including time-to-onset (TTO) from start of therapy until occurrence of suicidality, indication and applied dose at time of suicidality.
(iii) Personal history of suicide/suicidal ideation or self-injury.
(iv) Number of fatal suicide attempts.

Mean and median were calculated for patients’ age, the TTO from start of therapy and first occurrence of suidicality as well as the dose applied at the time of suicidality. For all other results, frequency distributions were calculated.

The completeness of each ADR report was assessed by calculating a completeness score using VigiGrade (mean calculated score: 0.89 [range: 0.28–1.0]; per definition a VigiGrade score ≥ 0.8 is designated as well-documented). The score was modified as the completeness of the ADR reports was only assessed for the reported ADR describing suicidality.

Reporting rates were calculated for the post-warning period (2009–2019) by dividing the number of confirmed reports from Germany for the individual drugs by their number of prescriptions. An analyses of reporting rates seperated by sex and age groups (12–18 years, and 19–24 years) were performed. It has to be considered that the calculation of reporting rates referred to a small number of confirmed reports from Germany. Reporting rates were only calculated for the drugs where the number of confirmed reports from Germany exceeded 3 reports.

## Results

### US and EU: identified reports and annual number of reports

In the analyzed period of time, 1,630 and 1,173 ADR reports referring to intentional suicide/suicidal behaviour or overdose for patients younger than 25 years were identified in FAERS (US) and EudraVigilance (EU) respectivly (ratio US/EU reports: 1.4). In the 1,630 US reports, fluoxetine (*n* = 475, 29.1%) was most frequently reported as suspected, followed by paroxetine (*n* = 376, 23.1%) and citalopram (*n* = 333, 20.4%). In contrast, sertraline ranked first in EU reports (*n* = 310, 26.5%), followed by fluoxetine (*n* = 286, 24.4%), citalopram (*n* = 217, 18.5%), escitalopram (*n* = 214, 18.3%), paroxetine (*n* = 133, 11.3%) and fluvoxamine (*n* = 34; 2.9%) (Table S1, available online).

**Fig. 2:**
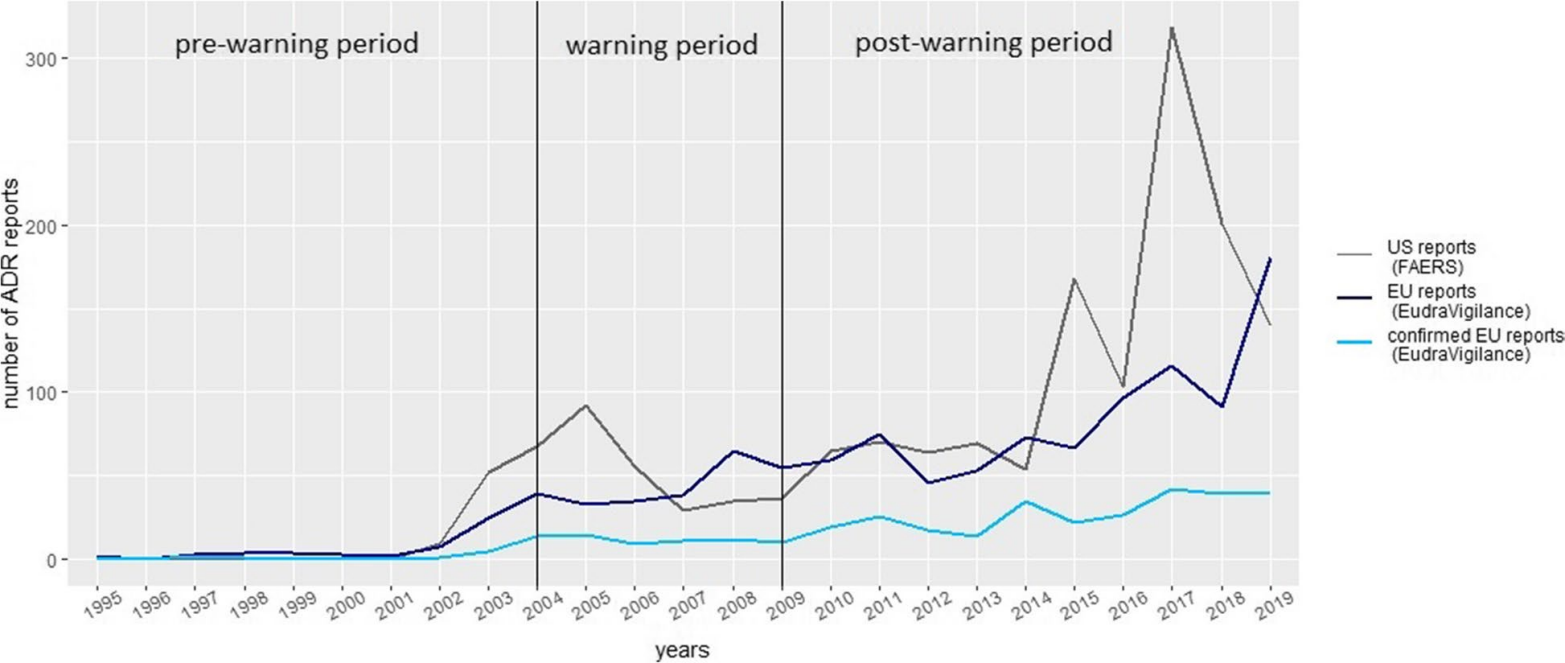
Annual number of reports from EudraVigilance and FAERS referring to intentional suicide/suicidal behaviour or overdose associated with SSRI. *Legend to Fig. 2:* EEA: European Economic Area; Confirmed EEA reports: reports from the European economic area with a possible causal relationship of suicidality related to SSRI treatment following causality assessment applying WHO criteria [20]. US: United States of America, FAERS: Food and Drug Administration (FDA) Adverse Event Reporting System

Before 2003 (pre warning period) only a few cases were reported in the US (*n* = 61) and the EU (*n* = 50). After 2003 the number of ADR reports referring to intentional suicide/suicidal behaviour or overdose for patients younger than 25 years and SSRI-therapy increased in the warning-period and, at a higher rate since 2014 in the post-warning period (see Fig. 2).

Several peaks of reporting numbers were observed that seemed to be related to specific drugs. In the US reports, paroxetine accounted to a large extent for the peaks between 2004–2005 (warning period), while in 2015 fluoxetine, citalopram, sertraline and paroxetine, and in 2017 fluoxetine, sertraline and citalopram were more often reported (Figure S1a, available online). In comparison only minor peaks in 2004 (paroxetine), 2008 (citalopram), 2011 (escitalopram, fluoxetine), 2014 (sertraline, fluoxetine) and 2017 (sertraline, fluoxetine) were observed for reports from the EU (Figure S1b, available online).

The causal relationship was assessed as at least possible in 362 of the 1,173 identified EU reports (from here on called “confirmed EU reports”). The number of confirmed EU reports did not exceed 50 reports per year and slightly increased in the post-warning period (Fig. 2).

**Fig. 3:**
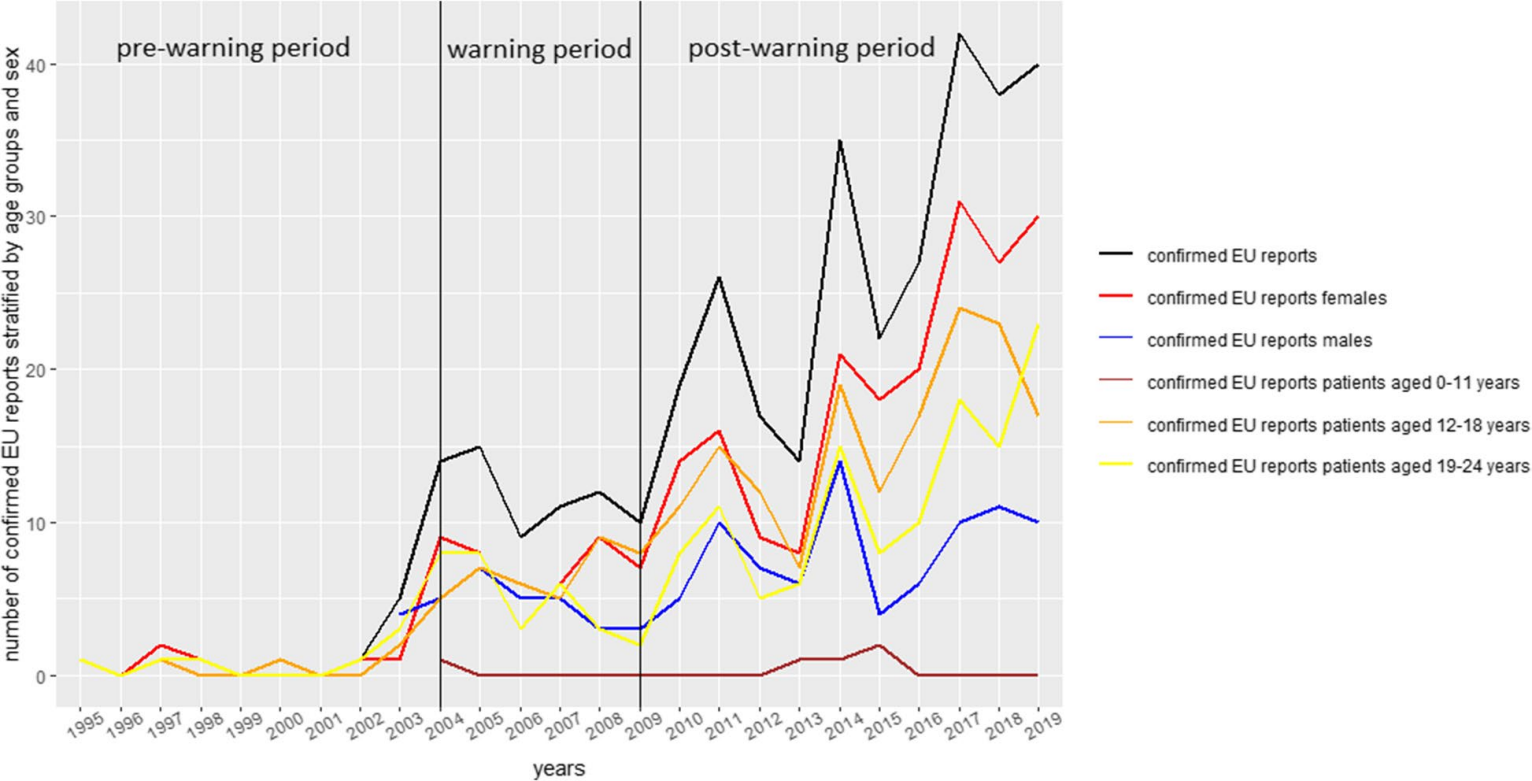
Annual number of confirmed selective serotonin reuptake inhibitor (SSRI) reports from Germany divided by sex and age groups

The analysis of the annual number of confirmed EU reports by sex and age groups showed that the slight increase was related to reports referring to females and 12–24 year olds (see Fig. 3).

Only single cases were reported for 0–11 year olds. However, comparing the number of SSRI reports reporting any other ADR (except for reports referring to suicidality) contained in EudraVigilance, an annual increase was also seen for patients 0–11 years (Data S1, available online).

### EU: Confirmed reports – in depth analysis

In the confirmed EU reports, the same ranking order for the SSRIs was observed (sertraline (*n* = 104), fluoxetine (*n* = 103), citalopram (*n* = 64), escitalopram (*n* = 61), paroxetine (*n* = 27), fluvoxamine (*n* = 4)) (Table S1, available online). Most of the confirmed EU reports were from the UK (*n* = 96, 25.5%), followed by Germany (*n* = 46, 12.7%), France (*n* = 38, 10.5%), Netherlands (*n* = 37, 10.2%) and Sweden (*n* = 35, 9.7%; see Data S2, available online).

Most of the confirmed reports were received in the post-warning period (*n* = 290, 80.1%) (different time intervals have to be considered; see Table 1).

**Table 1:** Analyses of characteristics reported in the pre-warning, warning and post-warning period

	Pre-warning period 1978–2003	Warning period 2004–2008	Post-warning period 2009–2019
Number of confirmed reports referring to suicidality^a^	11 (3.0%)	61 (16.9%)	290 (80.1%)
Demographic parameters of the patients			
Mean age	19.6	18.5	18.0
Median age	20.0	18.0	18.0
Female	63.6% (*n* = 7)	57.4% (*n* = 35)	69.3% (*n* = 201)
Male	36.4% (*n* = 4)	41.0% (*n* = 25)	29.7% (*n* = 86)
Unknown	0.0% (*n* = 0)	1.6% (*n* = 1)	1.0% (*n* = 3)
Reported type of suicidality^b^			
Suicidal ideation	18.2% (*n* = 2)	41.0% (*n* = 25)	52.4% (*n* = 152)
Suicide attempt	81.8% (*n* = 9)	52.5% (*n* = 32)	46.6% (*n* = 135)
Self-injury	0.0% (*n* = 0)	9.8% (*n* = 6)	14.8% (*n* = 43)
Overdose	0.0% (*n* = 0)	8.2% (*n* = 5)	8.3% (*n* = 24)
Median time to onset of suicidality [in days]^c^			
Suicidal ideation	62.5	20.5	11.0
Suicide attempt	24.5	23.0	31.0
Drugs reported as suspected with their median dose at time of reaction [in mg]^d^			
Citalopram	18.2% (*n* = 2), 25 mg	16.4% (*n* = 10), 20 mg	17.9% (*n* = 52), 20 mg
Escitalopram	9.1% (*n* = 1), 10 mg	19.7% (*n* = 12), 10 mg	16.6% (*n* = 48), 10 mg
Fluoxetine	45.5% (*n* = 5), 20 mg	31.1% (*n* = 19), 20 mg	27.6% (*n* = 80), 20 mg
Paroxetine	18.2% (*n* = 2), 10 mg	16.4% (*n* = 10), 20 mg	5.2% (*n* = 15), 20 mg
Sertraline	9.1% (*n* = 1), 20 mg	14.8% (*n* = 9), 50 mg	32.4% (*n* = 94), 50 mg
Fluvoxamine	0.0% (*n* = 0), -	1.6% (*n* = 1), 300 mg	1.0% (*n* = 3), 22.5 mg
Reported indication^e^			
Depression	81.8% (*n* = 9)	67.2% (*n* = 41)	64.1% (*n* = 186)
Anxiety disorder	18.2% (*n* = 2)	8.2% (*n* = 5)	14.1% (*n* = 41)
History of suicidality^f^			
Suicidal ideations	9.1% (*n* = 1)	11.5% (*n* = 7)	9.0% (*n* = 26)
Suicide attempts	18.2% (*n* = 2)	13.1% (*n* = 8)	7.9% (*n* = 23)
Self-injuries	0.0% (*n* = 0)	8.2% (*n* = 5)	5.5% (*n* = 16)
Seriousness of reports^g^			
Serious	100.0% (*n* = 11)	100.0% (*n* = 61)	95.2% (*n* = 276)
Death	36.4% (*n* = 4)	16.4% (*n* = 10)	13.1% (*n* = 38)
Life-threatening	9.1% (*n* = 1)	29.5% (*n* = 18)	26.2% (*n* = 76)
Hospitalization	36.4% (*n* = 4)	39.3% (*n* = 24)	37.2% (*n* = 108)
Disabling	9.1% (*n* = 1)	3.3% (*n* = 2)	6.9% (*n* = 20)
Primary reporting source^h^			
Physician	81.8% (*n* = 9)	65.6% (*n* = 40)	43.4% (*n* = 126)
Pharmacist	9.1% (*n* = 1)	6.6% (*n* = 4)	5.5% (*n* = 16)
Other healthcare professionals	9.1% (*n* = 1)	8.2% (*n* = 5)	7.2% (*n* = 21)
Patients	0.0% (*n* = 0)	8.2% (*n* = 5)	36.6% (*n* = 106)

^a^reports from the EU with an at least possible causal relationship between regular SSRI therapy and reported suicidality in accordance with the criteria of the WHO

^b^more than one of the respective events can be coded in one report

^c^median time to onset until first occurrence of suicidal ideations or suicides after initiation of SSRI therapy. The time to onset was not exactly provided in each report

^d^median dose at the time of suicidality. The dose was not provided in each report

^e^shown are the two indications most frequently reported

^f^one patient could have more than one event (e.g. suicidal ideations and suicide) in the past

^g^seriousness criteria in accordance with the German Drug Law

^h^shown are the number of reports referring to one primary reporting source

Patients in the post-warning period (median age of 18.0 years) were roughly two years younger than in the pre-warning period (median age of 20.0 years). The proportion of females was higher in the post-warning (69.3%) than in the pre- (63.6%) and the warning period (57.4%). The highest proportion of males was found in the warning period (41.0%).

The type of reported suicidality differed between the warning periods. Suicide attempts were more often reported in the pre-warning period (81.8%), while the number of reports referring to suicidal ideations, self-injuries and overdoses was higher in the warning and the post-warning period. In addition, the TTO between the intake of a SSRI to the occurrence of suicidal ideations decreased from the pre-warning to the post-warning period.

For the SSRIs reported as suspected, the number of reports for fluoxetine and paroxetine decreased from the pre- to post-warning period, while the number of reports for escitalopram and sertaline increased. An increase of the applied dose at the time of suicidality was observed for sertraline and paroxetine from the pre- to the post-warning period.

The reported indications in the warning and post-warning period were more diverse than in the pre-warning period. Compared to the warning period, a previous history of sucidial ideations, suicide attempts and self-injuries was proportionally less often reported in the post-warning period. Also, the proportion of fatal suicide attempts decreased from the pre- to the post-warning period.

An increase in the number of reports from patients was seen from the pre- to the post-warning period with regard to the reported characteristics of any other ADR (excl. suicidality) to SSRI (see Data S3, available online).

In the drug-stratified analysis the median TTO until occurrence of suicidal *ideation* was shortest for escitalopram (7.5 days) and longest for fluoxetine (19 days) (Table S2, available online). The median time-to-onset until first occurrence of suicide *attempts* was shortest for sertraline (25 days) and longest for fluoxetine (37 days) and citalopram (37 days).

Previous suicidal ideations (14.8%) were most common among the reports for escitalopram and previous suicide attempts among the reports for citalopram (14.1%) and escitalopram (13.1%).

In total, fatal suicide attempts were recorded in 52 reports (14.3%). Fatal suicide attempts were more often reported for fluoxetine (17.4%) and citalopram (17.2%) than for other SSRIs.

### Germany: Number of suicides, SSRI drug prescriptions and confirmed cases per year

In Germany, the number of children, adolescents, and young adults who commited suicide decreased steadily from 1996 to 2019 (Figure S2, available online), whereas SSRI prescriptions increased from 2009 onwards (Data S4, available online). For SSRI prescriptions, an increase of 133% was observed from 2009 to 2019 for patients 12–24 years. The increase was higher for patients 12–18 years (2009 to 2019: 243%) than for patients 19–24 years (2009 to 2019: 104%). Increases in SSRI prescriptions for females and males were similar (2009 to 2019 females + 134%, males: + 131%). From 2009 to 2019, the highest increase was seen for sertraline (+ 515%), followed by escitalopram (+ 378%) and fluoxetine (+ 158%). However, the number of confirmed reports from Germany remained constant in the post-warning period.

### Germany: Number of reports in relation to drug prescriptions

In the post-warning period (2009–2019), citalopram was the SSRI most often prescribed for patients between 12–24 years (*n* = 831,482), followed by fluoxetine (*n* = 670,432), escitalopram (*n* = 461,845) and sertaline ( *n* = 443,490; Table 2). For patients between 12–18 years, fluoxetine (*n* = 366,579), citalopram (*n* = 124,643) and sertraline (*n* = 100,283) and, for patients between 19–24 years citalopram (*n* = 706,839), escitalopram (*n* = 378,833) and sertraline (*n* = 343,207) were most frequently prescribed. The calculated reporting rate per 100,000 drug prescriptions of confirmed reports from Germany for patients between 12–24 years was highest for sertraline (2.0), followed by escitalopram (1.7), citalopram (1.0) and fluoxetine (0.9). When separated by age groups, the highest reporting rates were calculated for escitalopram (6.0) and citalopram (4.0) for patients between 12–18 years and, for sertaline (2.3) for patients between 19–24 years (see Table 2). Reporting rates for all SSRIs with the exception of fluoxetine were higher for females than for males.

**Table 2:** Number of confirmed selective serotonin reuptake inhibitor (SSRI) reports, number of SSRI prescriptions and the calculated reporting rates (number of confirmed SSRI reports/number of SSRI prescriptions) in the post-warning period from Germany

	Number of confirmed reports from Germany in post-warning period (2009–2019)	Number of drug prescriptions in post-warning period (2009–2019)	Reporting rate per 100,000 drug prescriptions in post-warning period (2009–2019)
SSRI	33	2,655,383	1.2
Female	26	1,738,983	1.5
Male	6	916,400	0.7
12–18 years	18	751,875	2.4
19–24 years	16	1,903,508	0.8
Citalopram	8	831,482	1.0
Female	7	541,856	1.3
Male	1	289,626	0.3
12–18 years	5	124,643	4.0
19–24 years	3	706,839	0.4
Escitalopram	8	461,845	1.7
Female	7	300,504	2.3
Male	1	161,341	0.6
12–18 years	5	83,012	6.0
19–24 years	3	378,833	0.8
Fluoxetine	7	670,432	1.0
Female	5	470,177	1.1
Male	2	200,255	1.0
12–18 years	6	366,579	1.6
19–24 years	1	303,853	0.3
Sertraline	9	443,490	2.0
Female	7	284,752	2.5
Male	2	158,738	1.3
12–18 years	1	223,337	0.4
19–24 years	8	343,207	2.3

1 reporting rates are calculated by the number of confirmed reports from Germany divided by the number of drug prescriptions

## Discussion

The aim of our study was to describe trends and patterns in spontaneous reporting data referring to suicidality in children, adolescents and young adults treated with SSRI after respective warnings.

### US and EU: annual number of reports

Our first new finding is that since the publication of the warnings, the number of spontaneous reports referring to suicide/suicidal ideations, intentional self-harm or overdoses for patients younger than 25 years has increased steadily in the US and the EU. The larger number of reports from the US compared to the EU might reflect the higher number of children and adolescents being treated with SSRIs in the US compared to the EU [23]. As also seen in prior analyses shortly before the warnings [5, 6], the increased number of the US and EU reports in the warning period in our analysis may be related to the media attention dedicated to the reports [24] and warnings themselves [1, 2].

A further new finding of our study is that the number of reports increased again in the post-warning period for the US and the EU. Several reasons might account for this oberservation such as, the higher level of attention to these adverse drug reactions due to the warnings, the increasing awareness of the existence and role of ADR reporting systems, the possibility to report online (especially for patients) [25], the widening of the ADR definition to include suicidal behaviors and overdoses since 2012 (EU) [18] and the increase of SSRI prescriptions [26–28]. The observed increase in the confirmed EU reports was less pronounced. For some of the countries (UK [26, 27], Sweden [29, 30], France [31], Germany [11, 12, 26] and Netherland [26]) most frequently reported in the confirmed EU reports (see Data S2, available online) an increase of SSRI prescriptions in the post-warning period was previously observed. Similar to our analysis of confirmed EU reports, the increase of SSRI prescriptions was mainly observed in adolescents, while the number of SSRI prescriptions for children remained stable or decreased, for example in France [31] and Germany [11, 12]. In our analysis, other ADRs to SSRIs seemd to be more relevant for 0–11 year olds or suicidality is more difficult to detect in 0–11 year olds and, thus, not reported [32].

### US and EU: Annual number of reports separated by drug

When comparing different SSRIs, differences in prescription patterns, revised guideline recommendations or increased attention due to warnings to specific SSRIs may have impacted on the time course of their number of reports. As an example, paroxetine was often prescribed for juvenile depression before 2004 [33, 34] and reported in the black-box warnings in 2004 [35]. This might explain the higher number of reports for paroxetine between 2001–2005 (Fig. 2; Figure S1a, available online). In accordance with the guidelines [36], fluoxetine has been recommended as a first line treatment for moderate to severe juvenile depression since 2005. Citalopram and sertralin are listed as second line options. Subsequently, their prescriptions [37] increased. Likewise, the number of reports for fluoxetine, sertraline, and citalopram in our analysis increased for the US and the EU.

Despite the similar guideline recommendations [38, 39], the observed differences in prescribing patterns in the US [26, 28, 34] and the EU [26, 34] as well as between EU countries [26], might explain the differences in the ranking of the most frequently reported SSRIs between the US and the EU reports in our analysis. Exact prescription data for the US and the EU are not available and assumptions based on published literature are complicated due to differences in study designs (e.g. analyzed period of time and age of the analyzed population). As the number of reports might be influenced by the number of prescriptions [40], we addressed this issue by considering the number of reports from Germany in relation to the number of SSRI prescriptions from Germany (see below).

In summary, there are several factors (e.g. warnings, higher media attention, changes in guidelines) which might have impacted on the number of reports. Hence, we cannot conclude that the increase is related to one single effect. Possibly, several factors have contributed to the observed patterns.

### EU: In-depth analysis of ADR reports referring to suicidality

The higher proportion of females in the confirmed EU reports may reflect more SSRI prescriptions [26, 27, 29] and diagnoses of juvenile depression and anxiety disorder [41] in females compared to males.

A more common ‘off-label’ use of SSRIs in the last few years [42] could explain the observed differences in reported indications between the post- and the pre-warning period. The higher proportion of patients with depressive disorders in the confirmed EU reports might be associated with an intrinsically increased risk of suicides in patients with depressive disorders [43] or more patients with depression being treated with SSRIs as opposed to other mental disorders. The link between SSRI and Selective Norepinephrine Reuptake Inhibitor (SNRI) and suicidality was more often associated with depressive disorders in a metaanalysis of 22 short-term placebo controlled trials [4]. In contrast, a lower effect was found for anxiety disorders [4]. It has to be taken into account that Wohlfarth and collegues [4] did not differentiate between SSRI and SNRI whereas our study referrs to SSRIs only.

It seems to be clinically plausible that suicidal ideations precede suicidal behaviors, as seen in our analysis (warning and post-warning period). Suicidal ideations and behaviors occurred mostly in the first three months of SSRI therapy. Some other studies also found a higher risk of suicidality after initiation of SSRI therapy [44, 45] whereas others did not [13]. Differences in the study designs may account for these inconsistencies. From a pharmacological perspective, it is assumed that in children and adolescents a delayed or insufficient mood improvement and early activating ADRs, like agitative symptoms, may lead to suicidal impulses after initiation of SSRI therapy. However, Näslund and collegues (2018) explored the effect of SSRIs on the item ‘suicidality’ of the Hamilton Rating Scale for Depression in young adults (18–24 years) and found a rapid, but weak, positive effect on suicidality and no indication of any subset of patients responding with an aggravation of suicidal thoughts [46]. Finally, the recommendation of a careful clinical monitoring especially in the first three months of SSRI therapy is supported by our results. 

It was striking that the proportion of reports referring to suicidal ideations increased from the pre- to the post-warning period in our analysis while the proportion of suicide attemps and fatal suicide attempts decreased. In addition, the TTO for suicidal ideations as given in the reports declined. It is possible that the suicidal ideations were recognized earlier after the warnings arised and led to a prevention of subsequent suicides. However, based on our analysis, we can not evaluate if children and adolescents treated with SSRI were monitored as recommended. Furthermore, a reporting bias of more serious reactions in the pre-warning period cannot be excluded. In contrast to our analysis, no fatal suicide attempts related to SSRIs were reported in RCTs [47, 48]. These discrepancies between the results of our analysis and RCTs might be explained by the exclusion of seriously ill, suicidal patients in RCTs.

In our analysis, only a few reports provided information with regard to the up-titration of SSRIs in the initial phase of SSRI therapy. Thus, we can not evaluate if SSRI dosages were slowly increased as recommended [36]. In our analysis, only the median dose for fluoxetine (20 mg/day)—but not for any other SSRI – mostly complied with the target (10-20 mg/day) than the initial dose (5(-10 mg)) at the time of sucidality.

9.4% and 9.1% of the patients had previous suicidal ideations or attempts. These patients might carry a higher risk of suidicality to SSRIs, as reported in literature [49]. Our analysis is consistent with these observations as prior suicidal ideations or attempts were much less reported in any other ADR reports to SSRIs compared to reports referring to suicidality (see Data S1, available online). It is possible that prior suicidal ideations or attempts in the any other ADRs data set may have been undetected as this analysis was based on coded events in the history of the patients, only, while the confirmed EU reports were assessed individually. The higher proportion of these patients in reports referring to suicidality for citalopram and escitalopram compared to other SSRIs might reflect a more severe course of disease. Citalopram is recommended as second line treatment if treatment with fluoxetine fails (EU), while escitalopram is not mentioned in the european guidelines [36] in these patients. Both SSRIs may be used off-label in the EU [50]. The decrease of the proportion of patients with a prior history of suicidal ideations or attempts from the pre- to the post-warning period might be related to a decrease of these patients being treated with SSRIs or a general decline of suicide attempts as fatal suicide attempts decreased in the analyzed period of time in some EU countries [51] including Germany.

### Germany: Number of suicides, SSRIs drug prescriptions and confirmed cases per year

As observed in the US [8, 52], an inverse relationship of increasing numbers of SSRI prescriptions and decreasing numbers of suicides was seen in the German data. Although the causal relationship cannot be assessed here, the increasing SSRI prescriptions do not seem to be associated with more suicides. Despite the increasing SSRI prescriptions between 2009–2019, the number of confirmed reports from Germany was stable (e.g. 139,000 drug prescriptions and 2 confirmed cases in 2009, and 309,000 drug prescriptions and 3 confirmed cases in 2018).

### Germany: Reporting rates of confirmed reports from Germany per 100,000 SSRI prescriptions

In our analysis, reporting rates differed with regard to individual SSRI, age groups and sex. For patients between 12–24 years, the highest reporting rate was calculated for sertraline, followed by escitalopram, citalopram and fluoxetine. Some studies found no differences between the SSRIs [48] whereas others reported differences for patients with anxiety disorders with a higher risk of suicidality for paroxetine compared to sertraline [53]. Others discussed if shorter half-lives of some SSRIs may play a role in the occurrence of suicidality [54]. In this respect, the reporting rate for sertraline, which has a short half-live (≅ 26 h), was higher than for fluoxetine (half-live ≅ 5 days) in our analysis. However, since there are no substantial differences in pharmacokinetics between citalopram and escitalopram the observed differences between their reporting rates might be by chance or related to differences in treated patient populations (e.g. co-morbidities). Based on our results, a higher risk for 12–18 year olds compared to 19–24 year olds, as well as for females compared to males might be assumed. In addition, drug-specific differences between age groups might be assumed. However, this difference may indicate a higher vigilance for suicidality in children and adolescents being treated ‘off-label’ or reflect a higher proportion of more severe course of disease in 12–18 year olds being treated with citalopram or escitalopram. It has to be taken into account that the number of confirmed reports from Germany was low, thus, the observed differences may also be by chance.

### Strengths and limitations

Strengths of our analysis include the coverage of a long period of time, the inclusion of a wide pediatric population including patients with severe depression or suicidal ideations and attempts in the past. Furthermore, external data sources such as the number of drug prescriptions and the number of suicides in the German population were considered in order to put the results into context.

One further advantage was the individual case assessment of the reports from the EU. Unfortunately, the causal association with the SSRI therapy was assessed as ‘at least possible’ in just 30.9% of the reports. Information about regular SSRI therapy was often not sufficiently documented.

One limitation of the study is the unknown amount of under-reporting that may also differ between the individual SSRIs. It seems plausible that the amount of under-reporting could be even higher before the widening of the ADR definition in 2012. On the other hand, the black box warnings may have stimulated the number of reports referring to suicidal ideations and behaviours to SSRIs.

Unfortunately, drug prescription data from all EU countries were not available and drug prescription data from Germany could only be provided from 2009. Furthermore, the number of drugs prescribed may not reflect the number of drugs actually used and refer only to patients with statutory health insurance (approximately 80–90% of inhabitants in Germany) and outpatient treatment.

## Conclusions

It may be that the increase in the number of US and EU reports was triggered by the media publicity of the reports and regulatory warnings in 2003–2005. Among other explanations, increased awareness due to the warnings or increases of drug prescriptions may have been responsible for a further increase in the number of reports from the US and the EU in the post-warning period. This result underlines the importance to monitor suicidality in children, adolescents and young adults treated with SSRIs, especially in the first three months after the start of SSRI therapy. If drug-associated suicidality is assumed, the case should be reported to the regulatory authorities with all relevant information thereby increasing the available data for future evaluations.

## Supplementary Information

**Additional file 1: Supplement Data 1.** Analysis of annual number of other ADR reports to SSRI.

**Additional file 2: Supplement Data 2.** Analysis of confirmed reports for the six most frequently reported EU countries.

**Additional file 3: Supplement Data 3.** Analysis of reported characteristics in the pre-warning, warning and post warning period of all other ADR reports to SSRI.

**Additional file 4: Supplement Data 4.** Number of SSRI prescriptions for patients 12-24 years in Germany in the post-warning period.

**Additional file 5: Figure S1a.** Annual number of reports per SSRI from the US. Figure S1a shows the annual number of reports per SSRI in the pre-warning, warning, and post-warning period from the US.

**Additional file 6: Figure S1b.** Annual number of reports per SSRI from the EU. Figure S1b shows the annual number of reports per SSRI in the pre-warning, warning, and post-warning period for the reports from the EU.

**Additional file 7: Figure S2.** Annual numbers of suicides of persons younger than 25 years in Germany.

**Additional file 8: Supplement Table 1.** SSRI most frequently reported as suspected in US, EU and confirmed EU reports.

**Additional file 9: Supplement Table 2.** Reported characteristics divided by Selective Serotonine Reuptake Inhibitors (SSRI).

## Data Availability

The data set generated and/or analysed during the current study are not publicly available due data privacy requirements. For further information regarding the processing of personal data in the context of the operation of EudraVigilance Human we refer to the European Medicines Agency’s Data Protection Notice for EudraVigilance Human (https://www.ema.europa.eu/en/documents/other/european-medicines-agencys-data-protection-notice-eudravigilance-human-ev_en.pdf). Researchers and/or readers who are interested can perform the same analyses in the ADR database EudraVigilance of the European Medicines Agency (EMA) (public access: http://www.adrreports.eu/en/index.html). However, different levels of access are granted for different stakeholders. The data that support the findings of this study are available from the corresponding author upon reasonable request.

## Abbreviations

ADR: Adverse Drug Reaction
BfArM: Federal Institute for Drugs and Medical Devices
EEA: European Economic Area
EU: European Union
FDA: Food and Drug Administration
FAERS: FDA Adverse Event Reporting System
G-BA: German innovation fund of the Federal Joint Committee
RCT: Randomized controlled trials
SNRI: Selective Norepinephrine Reuptake Inhibitor
SSRI: Selective Serotonin Reuptake Inhibitor
TTO: Time-to-onset
UK: United Kingdom
US: United States
WHO: World Health Organization
