# Supplementary material for: Selective serotonin reuptake inhibitors and suicidality in children and young adults: analyses of pharmacovigilance databases
Source: BMC Pharmacol Toxicol. 2023 Mar 31;24:22. doi: 10.1186/s40360-023-00664-z (PMC10067298; doi:10.1186/s40360-023-00664-z)
Supplement: Supplementary file 1 — Additional file 1: Supplement Data 1. Analysis of annual number of other ADR reports to SSRI. [file 40360_2023_664_MOESM1_ESM.docx]

Supplement Data 1: Analysis of annual number of other ADR reports to SSRI.

Introduction:

The aim was to analyse if there are differences in the annual number of other ADR reports (excl. suicidality) to SSRI compared to the reports referring to suicidality in the period investigated.

Method:

We extracted all spontaneous reports referring to the analysed SSRIs from the EU for patients 0-24 years which were received between 1978-2019. Afterwards, we excluded the 1,173 identified EU reports referring to intentional suicide/suicidal behaviour or overdose (n= 5,236).

Results:

Figure 1 Supplement Data 1. Annual number of other ADR reports to SSRIs divided by age groups and sex.


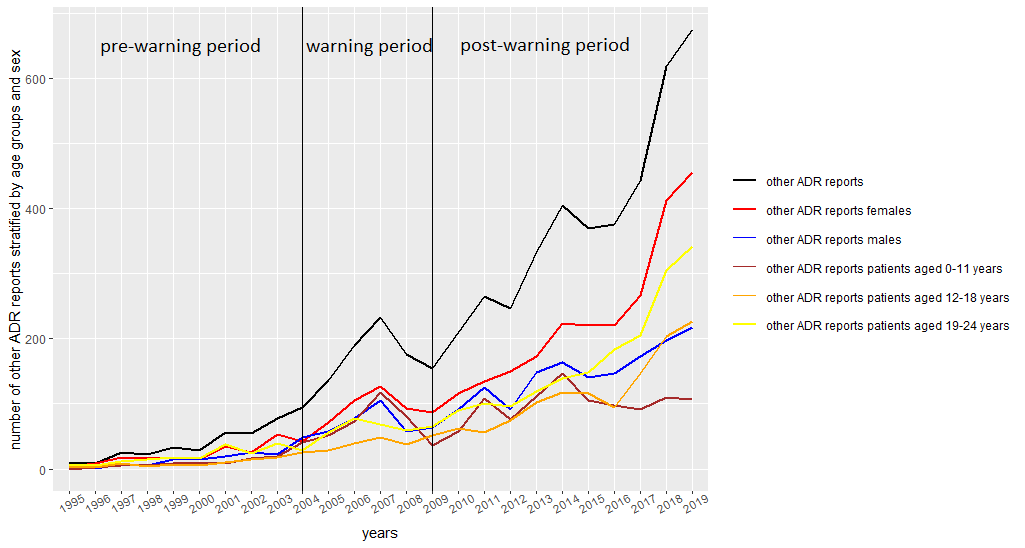


Legend Figure 1 Supplement Data 1:

Figure 1 Supplement Data 1 shows the annual number of reports referring to all other ADRs in which SSRIs were reported as suspected/interacting divided by sex and age groups.

Description:

The number of other ADR reports to SSRIs slightly increased in the pre-warning period. A larger increase was observed in the warning period, especially between 2004-2007. Then the number of other ADR reports decreased until 2009 but increased again in the post-warning period. A larger increase was observed for reports referring to females and to patients 19-24 years. However, also the number of reports for males and patients 0-18 years increased.

In comparison to the confirmed reports referring to suicidality, no peaks were observed in the early warning period (2004-2005) but the number of reports increased constantly in the early warning period. Likewise as for the confirmed reports referring to suicidality a peak was observed in 2007 with a slight decline of the number of reports until 2009. In the post-warning period, both the annual number of confirmed reports referring to suicidality and the number of other ADR reports increased again. The increase of the number of other ADR reports was also related to a larger increase of reports referring to females, however, also the reports for males and for patients 0-11 years increased which was not the case in the confirmed reported referring to suicidality.

Conclusion:

The higher increase of other ADR reports for females may be related to a higher increase of drug prescriptions for females than for males in the EU. Considering the number of drug prescriptions from Germany, the increase was rather equal for females and males in the post-warning period. If this would also apply to the EU prescriptions, a higher increase of suicidality and other ADRs to SSRIs for females in the post-warning period can be assumed. Controversy to our observations of only single reports referring to patients 0-11 years, the number of other ADR reports for patients 0-11 years increased in the period investigated. Thus, based on our results, a lower risk of suidicality to SSRI for patients 0-11 years compared to other ADRs can be assumed.
