# Supplementary material for: Selective serotonin reuptake inhibitors and suicidality in children and young adults: analyses of pharmacovigilance databases
Source: BMC Pharmacol Toxicol. 2023 Mar 31;24:22. doi: 10.1186/s40360-023-00664-z (PMC10067298; doi:10.1186/s40360-023-00664-z)
Supplement: Supplementary file 2 — Additional file 2: Supplement Data 2. Analysis of confirmed reports for the six most frequently reported EU countries. [file 40360_2023_664_MOESM2_ESM.docx]

Supplement Data 2) Analysis of confirmed reports for the six most frequently reported EU countries.

Table 1 Supplement Data 2. Analysis of the most frequently reported countries in the confirmed EU reports.

| Most frequently reported countries in the confirmed EU reports | Number of confirmed reports |
| --- | --- |
| United Kingdom | 96 (26.5%) |
| Germany | 46 (12.7%) |
| France | 38 (10.5%) |
| Netherlands | 37 (10.2%) |
| Sweden | 35 (9.7%) |
| Denmark | 30 (8.3%) |

Legend Table 1 Supplement Data 2:

Table 1 Supplement Data 2 shows the number and the relative share of the confirmed reports from the five most frequently reported countries from the EU besides Germany.

Description:

Most of the confirmed EU reports were from the United Kingdom (n= 96, 26.5%), followed by Germany (n= 46, 12.7%), France (n= 38, 10.5%), Netherlands (n= 37, 10.3%), Sweden (n= 35, 9.7%) and Denmark (n= 30, 8.3%).

Figure 1 Supplement Data 2. Stratified analysis of confirmed reports per year per country.


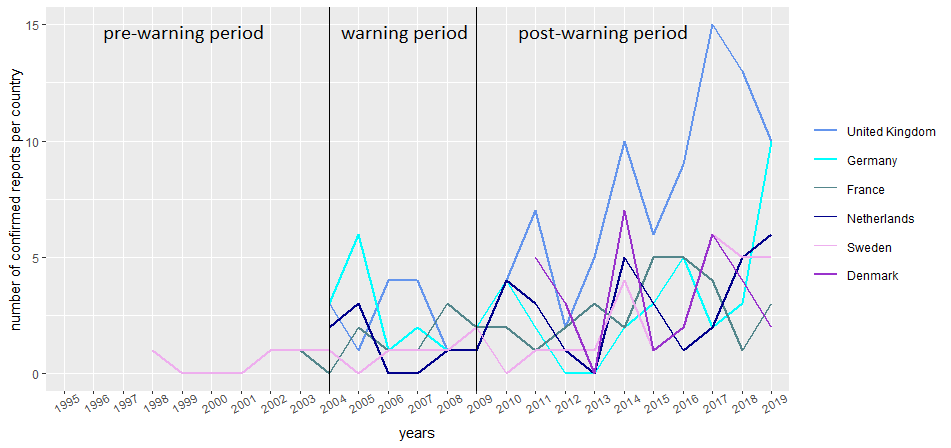


Legend Figure 1 Supplement Data 2:

Figure 1 Supplement Data 2 shows the number of confirmed reports for the five most frequently reported countries from the EU besides Germany.

Description:

Of the confirmed EU reports only single cases were reported before the warning period. In the warning period peaks were seen for the United Kingdom (2006-2007), Germany (2005 and 2007), Netherlands (2005) and France (2005 and 2008). For Sweden the number of confirmed reports was constantly low in the warning period. In the post-warning period, a slight increase of the number of confirmed reports from the United Kingdom, Germany, France, Netherlands and Sweden was seen. It has to be considered that the number of confirmed reports was still low.
