# Supplementary material for: Selective serotonin reuptake inhibitors and suicidality in children and young adults: analyses of pharmacovigilance databases
Source: BMC Pharmacol Toxicol. 2023 Mar 31;24:22. doi: 10.1186/s40360-023-00664-z (PMC10067298; doi:10.1186/s40360-023-00664-z)
Supplement: Supplementary file 3 — Additional file 3: Supplement Data 3. Analysis of reported characteristics in the pre-warning, warning and post warning period of all other ADR reports to SSRI. [file 40360_2023_664_MOESM3_ESM.docx]

Supplement Data 3) Analysis of reported characteristics in the pre-warning, warning and post warning period of all other ADR reports to SSRI.

Introduction:

The aim was to analyse if there are differences in the characteristics reported in other ADR reports (excl. suicidality) to SSRI compared to the reports referring to suicidality in the period investigated.

Method:

We extracted all spontaneous reports referring to the analysed SSRIs from the EU for patients 0-24 years which were received between 1978-2019. Afterwards, we excluded the 1,173 identified EU reports referring to intentional suicide/suicidal behaviour or overdose (n= 5,236).

Results:

Table 1 Supplement Data 3. Reported characteristics in the pre-warning, warning and post-warning period of all other ADR reports to SSRI-

|  | Pre-warning period 1978-2003 | Warning period 2004-2008 | Post-warning period 2009-2019 |
| --- | --- | --- | --- |
| Number of reports | 315 (6.0%) | 829 (15.8%) | 4,092 (78.2%) |
| Demographic parameters of the patients | | | |
| Mean age  Median age  Female  Male  Unknown | 15.8  19.0  60.8% (n= 192)  36.7% (n= 116)  2.2% (n= 7) | 11.1  15.0  52.7% (n= 437)  41.7% (n= 346)  5.5% (n= 46) | 14.7  17.0  61.0% (n= 2,458)  38.7% (n= 1,559)  1.9% (n= 75) |
| Most frequently reported ADRs (HLT) | | | |
| 1.  2.  3.  4.  5. | 7.6% seizures and seizure disorders (n= 24)  6.7% muscle tone abnormal (n= 21)  6.3% nausea and vomiting symptoms (n= 20)  6.0% disturbance in consciousness (n= 19)  4.4% rate and rhythm disorders (n= 14) | 11.6% exposures associated with pregnancy, delivery and lactation (n= 96)  8.8% withdrawal and rebound effects (n= 73)  6.9% seizure and seizure disorders (n= 57)  6.3% muscle tone abnormal (n= 52)  5.4% disturbance in consciousness (n= 45)  5.4% nausea and vomiting symptoms (n= 45) | 9.3% exposures associated with pregnancy, delivery and lactation (n= 380)  8.5% nausea and vomiting symptoms (n= 347)  7.2% disturbance in consciousness (n= 296)  6.6% neurological signs and symptoms (n= 270)  5.9% asthenic conditions (n= 240) |
| Drugs reported as suspected | | | |
| Citalopram  Escitalopram  Fluoxetine  Paroxetine  Sertraline  Fluvoxamine | 22.2% (n= 70)  1.3% (n= 4)  20.6% (n= 65)  32.7% (n= 103)  18.4% (n= 58)  5.4% (n= 17) | 19.8% (n= 164)  12.5% (n= 104)  21.8% (n= 181)  22.8% (n= 189)  20.0% (n= 166)  4.3% (n= 36) | 17.9% (n= 732)  16.2% (n= 663)  20.4% (n= 833)  10.6% (n= 432)  34.7% (n= 1,420)  1.9% (n= 76) |
| History of suicidality | | | |
| Suicidal and self-injurious behaviour | 0.3% (n= 1) | 0.8% (n= 7) | 2.6% (n= 105) |
| Seriousness of reports | | | |
| Serious  Death  Life-threatening  Hospitalisation  Disabling | 77.2% (n= 244)  3.8% (n= 12)  7.9% (n= 25)  48.1% (n= 152)  3.5% (n= 11) | 88.3% (n= 732)  3.9% (n= 32)  6.5% (n= 54)  40.0% (n= 332)  4.0% (n= 33) | 70.7% (n= 2,849)  2.1% (n= 86)  4.3% (n= 173)  25.7% (n= 1034)  4.4% (n= 178) |
| Primary reporting source | | | |
| Physician  Pharmacist  Other HCP  Consumer | 73.7% (n= 233)  1.6% (n= 5)  11.1% (n= 35)  1.3% (n= 4) | 66.2% (n= 549)  5.9% (n= 49)  10.0% (n= 83)  7.8% (n= 65) | 48.2% (n= 1,943)  8.4% (n= 338  8.1% (n= 326)  24.9% (n= 1,004) |

Description:

Patients with other ADRs were younger than patients with suicidality to SSRI. Likewise as for reports referring to suicidality the highest proportion of reports for males was also seen in the warning period. In the warning and the post-warning period almost one tenth of the reports referred to exposures during pregnancy, delivery or lactation. In contrast to reports referring to suicidality, there was no decrease for reports referring to fluoxetine for other ADRs between the three warning period. The proportion of patients with suicidal and self-injurious behaviour in the past was lower than in the reports referring to suicidality. In addition, the reports of other ADRs were less often serious, life-threatening or fatal. The proportion of reports from consumers was higher in reports referring to suicidality than in reports of other ADRs.

Conclusion:

The analysis of reported characteristics for other ADRs confirmed the assumption of a higher risk of suicidality to SSRI for patients 12-24 years than patients 0-11 years. It is unclear why there was a decrease of reports referring to suicidality for fluoxetine from the pre- to the post-warning period since this was not seen for other ADR reports. A previous history of suicidal and self-injurious behaviours may be more often associated with sucidality than with other ADRs to SSRI. Since consumer more frequently report ADR that are perceived more subjectively it seems plausible that the proportion of consumer reports was higher in reports referring to suicidality than to other ADRs.
