# Supplementary material for: Selective serotonin reuptake inhibitors and suicidality in children and young adults: analyses of pharmacovigilance databases
Source: BMC Pharmacol Toxicol. 2023 Mar 31;24:22. doi: 10.1186/s40360-023-00664-z (PMC10067298; doi:10.1186/s40360-023-00664-z)
Supplement: Supplementary file 4 — Additional file 4: Supplement Data 4. Number of SSRI prescriptions for patients 12-24 years in Germany in the post-warning period. [file 40360_2023_664_MOESM4_ESM.docx]

Supplement Data 4) Number of SSRI prescriptions for patients 12-24 years in Germany in the post-warning period.

Supplement Data 4 Figure 1. Annual number of SSRI prescriptions in Germany stratified by age groups and sex.


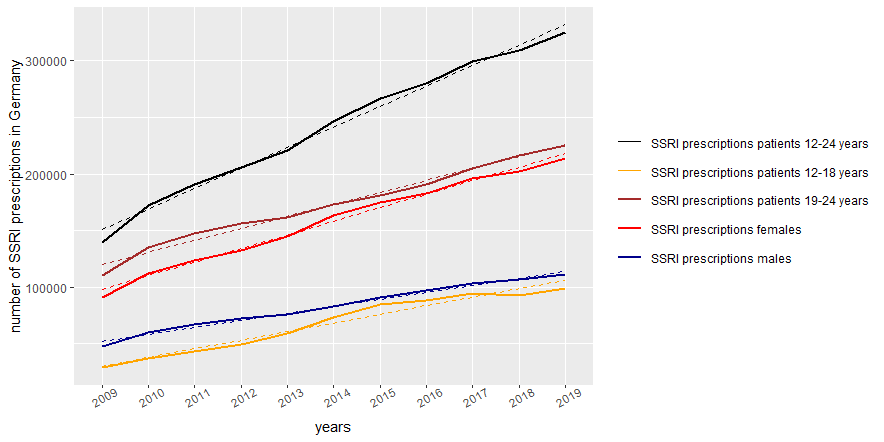


Legend:

Supplement Data 4 Table 1 shows the annual increase of the number of SSRI prescriptions for patients 12-24 years in Germany stratified by sex and age groups (12-18 years, 19-24 years).

Supplement Data 4 Table 1. Annual number of SSRI prescriptions in Germany stratified by age groups and sex and their yearly increase.

| Year | SSRI prescriptions patients 12-14 years (% increase to prior year) | SSRI prescriptions patients 12-18 years (% increase to prior year) | SSRI prescriptions patients 19-24 years (% increase to prior year) | Ratio patients aged  19-24/  12-18 years | SSRI prescriptions females (% increase to prior year) | SSRI prescriptions males (% increase to prior year) | Ratio females/males |
| --- | --- | --- | --- | --- | --- | --- | --- |
| 2009 | 139201 | 28846 | 110355 | 3.8 | 91211 | 47990 | 1.9 |
| 2010 | 172263 (23.8%) | 36872 (27.8%) | 135391 (22.7%) | 3.7 | 112268 (23.1%) | 59995 (25.0%) | 1.9 |
| 2011 | 190896 (10.8%) | 43382 (17.7%) | 147514 (9.0%) | 3.4 | 123976 (10.4%) | 66920 (11.5%) | 1.9 |
| 2012 | 205807 (7.8%) | 49561 (14.2%) | 156246 (5.9%) | 3.2 | 132923 (7.2%) | 72884 (8.9%) | 1.8 |
| 2013 | 220630 (7.2%) | 58823 (18.7%) | 161807 (3.6%) | 2.8 | 144718 (8.9%) | 75912 (4.2%) | 1.9 |
| 2014 | 246511 (11.7%) | 73566 (25.1%) | 172945 (6.9%) | 2.4 | 163376 (12.9%) | 83135(9.5%) | 2.0 |
| 2015 | 266448 (8.1%) | 85137 (15.7%) | 181311 (4.8%) | 2.1 | 175319 (7.3%) | 91129 (9.6%) | 1.9 |
| 2016 | 279789 (5.0%) | 88736 (4.2%) | 191053 (5.4%) | 2.2 | 182874 (4.3%) | 96915 (6.3%) | 1.9 |
| 2017 | 299763 (7.1%) | 94842 (6.9%) | 204921 (7.3%) | 2.2 | 196330 (7.4%) | 103433 (6.7%) | 1.9 |
| 2018 | 309436 (3.2%) | 93008 (-1.9%) | 216428 (5.6%) | 2.3 | 202300 (3.0%) | 107136 (3.6%) | 1.9 |
| 2019 | 324639 (4.9%) | 99102 (6.6%) | 225537 (4.2%) | 2.3 | 213688 (5.6%) | 110951 (3.6%) | 1.9 |
| % increase from 2009 to 2019 | +133.2% | +243.5% | +104.4% |  | +134.3% | +131.2% |  |

Legend:

Supplement Data 4 Table 1 shows the annual number of SSRI prescriptions for patients 12-24 years in Germany stratified by sex and age groups (12-18 years, 19-24 years). Further on, the yearly increases to the prior year and the ratios for patients 19-24 years/12-18 years and for females/males were calculated.

Description:

The number of SSRI prescriptions for patients aged 12-24 years increased in the post-warning period (2009 – 2019). There was an increase of SSRI prescription of +133.2% from 2009 to 2019 for patients 12-24 years. A larger increase was observed for patients aged 12-18 years (2009 to 2019: +243.5%) than for patients aged 19-24 years (2009 to 2019: +104.4%). Increases of SSRI prescriptions for females and males were rather equal (2009 to 2019 females: +134.3$, males: +131.2%). The calculated ratios also showed a larger increase for patients 12-18 years than for patients 19-24 years between 2009-2019, while the ratio for females/males was constant. In total, the number of SSRI prescriptions was higher for patients aged 19-24 years and for females compared to patients aged 12-18 years and to males.

Supplement Data 4 Figure 2) Annual number of SSRI prescriptions in Germany stratified by SSRI.


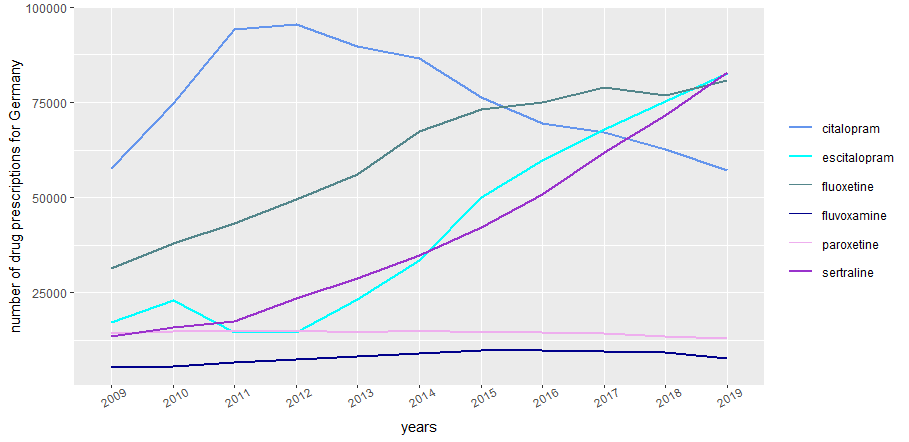


Legend:

Supplement Data 4 Figure 2 shows the annual number of SSRI prescriptions for patients aged 12-24 years for citalopram, escitalopram, fluoxetine, fluvoxamine, paroxetine and sertraline.

Supplement Data 4 Table 2) Annual number of SSRI prescriptions in Germany stratified by SSRI and their yearly increase.

| Year | Citalopram prescriptions (% increase to prior year) | Escitalopram prescriptions (% increase to prior year) | Fluoxetine prescriptions patients 19-24 years (% increase to prior year) | Fluvoxamine prescriptions (% increase to prior year) | Paroxetine prescriptions (% increase to prior year) | Sertraline prescriptions (% increase to prior year) |
| --- | --- | --- | --- | --- | --- | --- |
| 2009 | 57632 | 17275 | 31298 | 5292 | 14223 | 13481 |
| 2010 | 74762 (29.7%) | 23006 (33.2%) | 37994 (21.4%) | 5726 (8.2%) | 14970 (5.3%) | 15805 (17.2%) |
| 2011 | 94096 (25.9%) | 14635 (-36.4%) | 43225 (13.8%) | 6688 (16.8%) | 14823 (-1.0%) | 17429 (10.3%) |
| 2012 | 95495 (1.5%) | 14464 (-1.2%) | 49566 (14.7%) | 7619 (13.9%) | 15023 (1.3%) | 23640 (35.6%) |
| 2013 | 89714 (-6.1%) | 23165 (60.2%) | 56016 (13.0%) | 8241 (8.2%) | 14703 (-2.1%) | 28791 (21.8%) |
| 2014 | 86622 (-3.4%) | 33419 (44.3%) | 67373 (20.3%) | 9067 (10.0%) | 15160 (3.1%) | 34870 (21.1%) |
| 2015 | 76325 (-11.9%) | 50130 (50.0%) | 73269 (8.8%) | 9914 (9.3%) | 14650 (-3.4%) | 42160 (20.9%) |
| 2016 | 69594 (-8.8%) | 59826 (19.3%) | 75117 (2.5%) | 9820 (-0.9%) | 14540 (-0.8%) | 50892 (20.7%) |
| 2017 | 67253 (-3.4%) | 67861 (13.4%) | 78826 (4.9%) | 9683 (-1.4%) | 14305 (-1.6%) | 61835 (21.5%) |
| 2018 | 62785 (-6.6%) | 75354 (11.0%) | 76851 (-2.5%) | 9233 (-4.6%) | 13576 (-5.1%) | 71637 (15.9%) |
| 2019 | 57204 (-8.9%) | 82710 (9.8%) | 80897 (5.3%) | 7769 (-15.9%) | 13109 (-3.4%) | 82950 (15.8%) |
| % increase from 2009 to 2019 | -0.7% | +378.8% | +158.5% | +46.8% | -7.8% | +515.3% |

Supplement Data 4 Table 2 shows the annual number of SSRI prescriptions stratified by citalopram, escitalopram, fluoxetine, fluvoxamine, paroxetine and sertraline.

Description:

At the beginning of the post-warning period (2009-2012) the number of citalopram prescriptions increased following by a decrease after 2012. For fluoxetine, and sertraline an increase of prescriptions was observe in the post-warning period and for escitalopram from 2012 onwards. The number of paroxetine and fluvoxamine prescriptions was rather constant and was lower than for the other SSRI. In the post-warning period, the largest increase was observed for sertraline with +515.3% from 2009 to 2019, followed by escitalopram (2009 to 2019: +378.8%) and fluoxetine 2009 to 2019: +158.5%). A decrease of prescriptions from 2009 to 2019 was seen for citalopram (-0.7%) and paroxetine (-7.8%).
