# Supplementary material for: Selective serotonin reuptake inhibitors and suicidality in children and young adults: analyses of pharmacovigilance databases
Source: BMC Pharmacol Toxicol. 2023 Mar 31;24:22. doi: 10.1186/s40360-023-00664-z (PMC10067298; doi:10.1186/s40360-023-00664-z)
Supplement: Supplementary file 5 — Additional file 5: Figure S1a. Annual number of reports per SSRI from the US. Figure S1a shows the annual number of reports per SSRI in the pre-warning, warning, and post-warning period from the US. [file 40360_2023_664_MOESM5_ESM.docx]

Figure S1a. Annual number of reports per SSRI from the US.


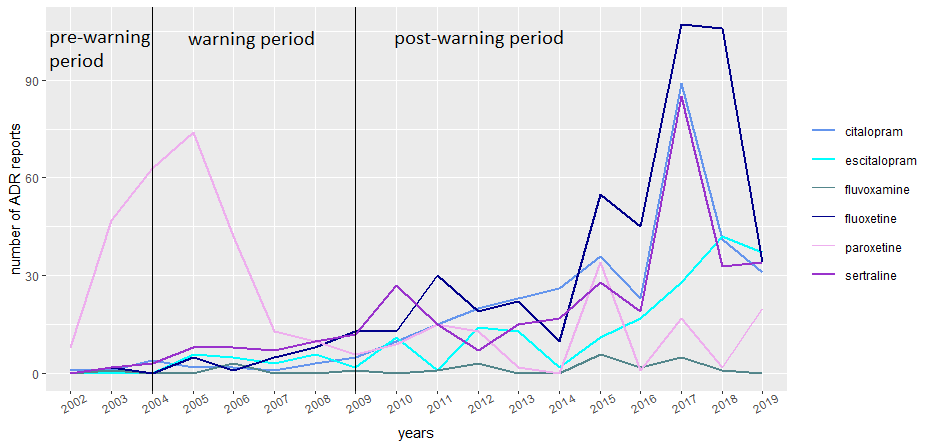


Figure S1a shows the annual number of reports per SSRI in the pre-warning, warning, and post-warning period from the US.
