# Supplementary material for: Selective serotonin reuptake inhibitors and suicidality in children and young adults: analyses of pharmacovigilance databases
Source: BMC Pharmacol Toxicol. 2023 Mar 31;24:22. doi: 10.1186/s40360-023-00664-z (PMC10067298; doi:10.1186/s40360-023-00664-z)
Supplement: Supplementary file 7 — Additional file 7: Figure S2. Annual numbers of suicides of persons younger than 25 years in Germany. [file 40360_2023_664_MOESM7_ESM.docx]

Figure S2. Annual numbers of suicides of persons younger than 25 years in Germany.


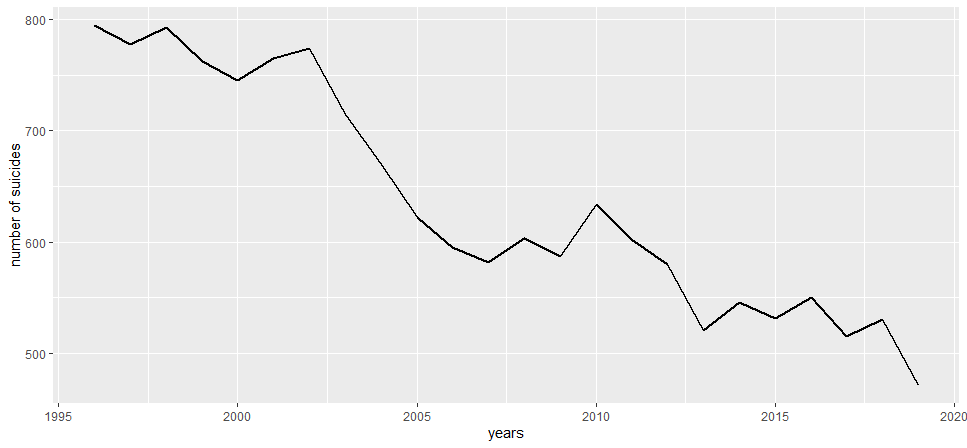


The annual numbers of suicides of persons younger than 25 years in Germany were extracted from the Federal Statistical Office in Germany.
