# Supplementary material for: Selective serotonin reuptake inhibitors and suicidality in children and young adults: analyses of pharmacovigilance databases
Source: BMC Pharmacol Toxicol. 2023 Mar 31;24:22. doi: 10.1186/s40360-023-00664-z (PMC10067298; doi:10.1186/s40360-023-00664-z)
Supplement: Supplementary file 8 — Additional file 8: Supplement Table 1. SSRI most frequently reported as suspected in US, EU and confirmed EU reports. [file 40360_2023_664_MOESM8_ESM.docx]

Supplement Table 1) SSRI most frequently reported as suspected in US, EU and confirmed EU reports.

|  | US reports (FAERS) (n= 1,630) | EU reports (EudraVigilance) (n= 1,173) | Confirmed EU reports (n= 362) |
| --- | --- | --- | --- |
| SSRI most frequently reported as suspected | | | |
| 1.  2.  3.  4.  5.  6. | fluoxetine (n= 475, 29.1%)  paroxetine (n= 376, 23.1%)  citalopram (n= 333, 20.4%)  sertraline (n= 330, 20.2%)  escitalopram (n= 198, 12.1%)  fluvoxamine (n= 23, 1.4%) | sertraline (n= 310, 26.4%)  fluoxetine (n= 286, 24.4%)  citalopram (n= 217, 18.5%)  escitalopram (n= 214, 18.2%)  paroxetine (n= 133, 11.3%)  fluvoxamine (n= 34, 2.9%) | sertraline (n= 104, 28.7%)  fluoxetine (n= 103, 28.5%)  citalopram (n= 64, 23.2%)  escitalopram (n= 61, 16.9%)  paroxetine (n= 27, 7.5%)  fluvoxamine (n= 4, 1.1%) |

FAERS = Food and Drug Administration (FDA) Adverse Event Reporting System, EudraVigilance = European ADR database, US = United States; EU = European Union
