# Supplementary material for: Selective serotonin reuptake inhibitors and suicidality in children and young adults: analyses of pharmacovigilance databases
Source: BMC Pharmacol Toxicol. 2023 Mar 31;24:22. doi: 10.1186/s40360-023-00664-z (PMC10067298; doi:10.1186/s40360-023-00664-z)
Supplement: Supplementary file 9 — Additional file 9: Supplement Table 2. Reported characteristics divided by Selective Serotonine Reuptake Inhibitors (SSRI). [file 40360_2023_664_MOESM9_ESM.docx]

Supplement Table 2) Reported characteristics divided by Selective Serotonine Reuptake Inhibitors (SSRI).

|  | Spontaneous confirmed reports^1^, all SSRIs (n= 362) | Spontaneous confirmed reports^1^, sertraline (n= 104) | Spontaneous confirmed reports^1^,  fluoxetine (n= 103) | Spontaneous confirmed reports^1^,  citalopram (n= 64) | Spontaneous confirmed reports^1^,  escitalopram (n= 61) |
| --- | --- | --- | --- | --- | --- |
| Demographic parameters  Female  Male  Unknown | 67.1% (n= 243)  31.7% (n= 115)  1.1% (n= 4) | 67.3% (n= 70)  32.7% (n= 34)  - | 68.0% (n= 70)  29.1% (n= 30)  2.9% (n= 3) | 67.2% (n= 43)  32.8% (n= 21)  - | 65.6% (n= 40)  34.4% (n= 21)  - |
| Drug indication | 65.2% depression (n= 236)  13.3% anxiety/panic disorder (n= 48) | 57.7% depression (n= 60)  18.3% anxiety/panic (n= 19) | 68.9% depression (n= 71)  7.8% anxiety (n= 8) | 64.0% depression (n= 41)  15.6% anxiety/panic disorder (n= 10) | 68.9% depression (n= 42)  9.8% anxiety/panic disorder (n= 6) |
| Suicidal ideation alone/ combination^3^  Suicide attempt alone/ combination^3^  Self-injurious behaviour alone/ combination^3^  Overdose alone/ combination^3^ | 36.5% (n= 132)/  49.4% (n= 179)  36.2% (n= 131)/  48.6% (n= 176)  5.8% (n= 21)/  13.5% (n= 49)  2.8% (n= 10)/  8.0% (n= 29) | 36.5% (n= 38)/  51.9% (n= 54)  34.6% (n= 36)/  50.0% (n= 52)  7.7% (n= 8)/  14.4% (n= 15)  1.9% (n= 2)/  5.8% (n= 6) | 34.0% (n= 35)/  46.6% (n= 48)  40.8% (n= 42)/  50.5% (n= 52)  5.8% (n= 6)/  14.6% (n= 15)  2.9% (n= 3)/  8.7% (n= 9) | 40.6% (n= 26)/  51.6% (n= 33)  31.3% (n= 20)/  45.3% (n= 29)  9.4% (n= 6)/  15.6% (n= 10)  1.6% (n= 1)/  6.3% (n= 4) | 34.0% (n= 20)/  45.9% (n= 28)  34.0% (n= 22)/  50.0% (n= 31)  2.0% (n= 2)/  10.0% (n= 7)  8.0% (n= 4)/  16.0% (n= 8) |
| Median dose (mg) | - | 50.0 | 20.0 | 20.0 | 10.0 |
| Median time to onset to suicidal ideation | 12.5 days | 8.0 days | 19 days | 11.5 days | 7.5 days |
| Median time to onset to suicide attempt | 30.0 days | 25.0 days | 37.0 days | 37.0 days | 30.0 days |
| Psychiatric history of suicidality ^4^ | 9.4% suicidal ideation (n= 34)  9.1% suicide attempt (n= 33)  5.8% self-injury (n= 21) | 11.5% suicidal ideation (n= 12)  7.7% suicide attempt (n= 8) 6.7% self-injury (n= 7) | 5.8% suicidal ideation (n= 6)  7.7% suicide attempt (n=8)  9.7% self-injury (n= 10) | 9.4% suicidal ideation (n= 6)  14.1% suicide attempt (n= 9) 6.3% self-injury (n= 43) | 14.8% suicidal ideation (n= 9)  13.1% suicide attempt (n= 8)  1.6% self-injury (n= 1) |
| fatal suicide attempts | 14.3% (n= 52) | 11.5% (n= 12) | 17.4% (n= 18) | 17.2% (n= 11) | 13.1% (n= 8) |

^ADR= Adverse Drug Reaction, SSRI= Selective Serotonine Reuptake Inhibitor^

^1^ confirmed ADR reports: ADR reports with a possible causal relationship in accordance with the criteria of the WHO^20^. Fluvoxamine (n= 2) and paroxetine (n= 1) were only rarely reported and are thus not presnted in Supplementary Table 2.

^2^ number of confirmed cases from Germany in the whole analysis periode

^3^ alone: only the respective event was coded; combination: more than one of the respective events was coded

^4^ multiple assignments are possible. One patient may have had suicidal ideations and suicide attempts in the past
